# Implementation of a large-scale simulation-based cardiovascular clinical examination course for undergraduate medical students – a pilot study

**DOI:** 10.1186/s12909-019-1750-4

**Published:** 2019-09-18

**Authors:** Dimitri Arangalage, Jérémie Abtan, Jean Gaschignard, Pierre-François Ceccaldi, Sid-Ahmed Remini, Isabelle Etienne, Philippe Ruszniewski, Patrick Plaisance, Victoire De Lastours, Agnès Lefort, Albert Faye

**Affiliations:** 1 Department of Cardiology, Bichat Hospital, AP-HP, 46 Rue Henri Huchard, 75018 Paris, France; 2 Department of General Pediatrics, Internal Medicine and Infectious Diseases, Robert Debré University Hospital, Paris, AP-HP France; 3 Department of Obstetrics and Gynecology, Beaujon Hospital, AP-HP, Clichy, France; 4 Department of Gastroenterology and Pancreatology, Beaujon Hospital, AP-HP, Clichy, France; 5 Department of Internal Medicine, Beaujon Hospital, AP-HP, Clichy, France; 6 Emergency Department, Lariboisière Hospital AP-HP, Paris, France; 7 Université de Paris, Faculté de Médecine Paris-Diderot, Paris, France; 8 iLumens Paris-Diderot Simulation Department, Paris, France; 9 INSERM U1148 (LVTS), Paris, France; 10 INSERM U1137 (IAME), Paris, France; 11 INSERM U1123 (ECEVE), Paris, France

**Keywords:** Clinical education, Mannequins, Simulation, Auscultation, Heart sounds

## Abstract

**Background:**

We report the implementation of a large-scale simulation-based cardiovascular diagnostics course for undergraduate medical students.

**Methods:**

A simulation-based course was integrated into the curriculum of second-year medical students (> 400 students/year). The first session aimed at teaching cardiac auscultation skills on mannequins and the second at teaching blood pressure measurement, peripheral arterial examination, and the clinical examination of heart failure in a technical skill-based manner and in a scenario.

**Results:**

A total of 414 (99.8%) and 402 (98.5%) students, as well as 102 and 104 educators, participated during the 2016–2017 and 2017–2018 academic years across both types of sessions. The number of positive appreciations by students was high and improved from the first to the second year (session 1: 77% vs. 98%, session 2: 89% vs. 98%; p < 0.0001). Similar results were observed for educators (session 1: 84% vs. 98%, p = 0.007; session 2: 82% vs. 98%, p = 0.01). Feedbacks by students were positive regarding the usefulness of the course, fulfillment of pedagogical objectives, quality of the teaching method, time management, and educator-student interactivity. In contrast, 95% of students criticized the quality of the mannequins during the first year leading to the replacement of the simulation material the following year. Students most appreciated the auscultation workshop (25%), the practical aspect of the course (22%), and the availability of educators (21%).

**Conclusions:**

Despite the need to commit significant human and material resources, the implementation of this large-scale program involving > 400 students/year was feasible, and students and educators reacted favorably.

## Background

The first year of medical studies in France is exclusively theoretical and students reach a real patient’s bedside during the second year. At our university, the teaching of clinical examination to second-year students was previously exclusively based on theoretical lectures and short bedside teaching sessions of 3 h a week in small groups. As the time allocated to bedside teaching had markedly declined during the past years at our university, as well as in most institutions worldwide [1–4], the development of alternative teaching methods had become an essential task. As a consequence, we implemented a simulation-based education program and a dedicated simulation center was created with the purpose of promoting simulation in healthcare education.

Simulation-based medical teaching has rapidly expanded over the past decade in the light of several studies that have consistently demonstrated its effectiveness in improving students’ skills and performance [5–12]. Although several studies have shown the benefits of such educational programs, large-scale implementations have seldom been reported [6–8, 13, 14], mainly because such programs represent a considerable investment, both in terms of financial and human resources.

In this article, we seek to describe the implementation and demonstrate the feasibility of a large-scale, compulsory, simulation-based cardiovascular diagnostics course for undergraduate medical students. Our main objective was to assess whether level 1 of Kirkpatrick’s model (evaluation of the degree of favorable reactions to learning events by participants) [15, 16] was reached by analyzing the perceptions of educators and undergraduate medical students.

## Methods

### Simulation-based cardiovascular diagnostics course description

The course was divided into 2 consecutive compulsory sessions of 75 min each aiming at teaching basic cardiovascular clinical examination skills to just over 400 students and took place at the iLumens Paris-Diderot platform (Université de Paris, Paris, France) dedicated to teaching through simulation. Our experience over 2 consecutive years is reported in the present study. Prior to the beginning of the course, a 10-min oral presentation was made to explain the educational objectives, followed by a 10-min video precisely showing each step of the cardiovascular clinical examination and reminding basic anatomy and physiology concepts (link to the video: https://youtu.be/MhfiDq2XePU). The objectives of the first session were to teach the examination and palpation of the precordium, followed by heart auscultation on mannequins using an electronic stethoscope, including normal and pathological heart sounds (Lifeform® Auscultation Trainer and Smartscope®). At the end of this session, 5 short clinical scenarios on previously taught valvular heart diseases (aortic stenosis, aortic regurgitation and mitral regurgitation) were used to evaluate students’ understanding. Each student was individually exposed to all 5 cases. These scenarios consisted of brief and typical case presentations after which students had to use the stethoscope on mannequins to diagnose and characterize valvular conditions. All scenarios were developed by cardiologists and approved by expert members of the pedagogical committee. The second session was aimed at teaching blood pressure measurement using a manual sphygmomanometer (Welch Allyn®), peripheral arterial auscultation as well as pulse localization and palpation, and finally the clinical examination of heart failure in a technical skill-based manner and considering communication with other students acting as simulated patients in a scenario.

All educators were junior staff attendings from various medical specialties, including non-cardiologists, provided with a detailed instruction manual describing how to use the mannequins, the educational objectives, and the duration of each step of the course. In addition, a senior cardiologist coordinating the education program and a technical supervisor demonstrated the operation of the simulation equipment to all educators before the course. The senior cardiologist and the technical supervisor were present on site during both sessions. Students were divided into small groups of no more than 4 students for 1 educator in order to facilitate educator-student interaction and to maximize the time spent practicing and using the teaching equipment. A total of 16 independent rooms were provided at the simulation platform, and therefore 16 educators and up to 64 students were simultaneously present at the simulation platform. Two administrative staff members were actively involved in the organization of the module including sessions scheduling, students’ registration, and student orientation at the platform. A total of 8 mannequins dedicated to cardiac auscultation were used, as half of the students attended the first session involving auscultation of mannequins, and the second half simultaneously attended the second session that did not require mannequins dedicated to auscultation. After 75 min, all students and teachers changed rooms to complete the other session.

### Students’ and educators’ perception evaluation tools

In order to assess the relevance of the teaching program, we used Kirkpatrick’s model of evaluation, which is divided into 4 levels - Level 1: evaluation of the degree of favorable reactions to learning events by participants; level 2: evaluation of knowledge acquisition (level 2a: attitudes/perceptions and level 2b: knowledge/skills); level 3: evaluation of behavioral changes and to what extent participants apply what they have learned during the training; and level 4: overall impact of training (level 4a: organizational practice, level 4b: student benefit and level 4c: patient benefit) [15, 16]. In the present study, we specifically evaluated whether the first level of Kirkpatrick’s model was reached. For this purpose, students and educators were asked to complete a course evaluation. The questionnaire was approved by the pedagogical committee of Paris-Diderot University, mandatory, anonymous and could be completed on a printed document or on any mobile device. The students’ evaluation consisted of 6 questions based on a score ranging from 0 to 3 (0 = poor, 1 = fair, 2 = good, 3 = excellent). These questions were ordered as follows: 1) usefulness of the course, 2) fulfillment of pedagogical objectives, 3) quality of the teaching method, 4) time management during the session, 5) educator-student interactivity and relationship, and 6) quality of the simulation equipment. In addition, a free response area was provided so students could specify the most and least useful aspects of the course and make suggestions for improvement. The educators’ survey consisted of 1 question assessing the overall satisfaction with the course based on the same score ranging from 0 to 3. The feedback was considered positive in case of a score ≥ 2.

### Statistical analysis

Continuous variables were expressed as mean ± standard deviation, or numbers (percentages). The Shapiro-Wilk test was used to evaluate distribution among variables. As continuous variables were not normally distributed in the present study, the Wilcoxon test was used for comparison between groups. The χ2 test was used for comparison between categorical variables. The total score presented in the students’ survey is defined by the average (mean ± standard deviation) of the 6 individual questions. The analysis of the free response area is presented as the percentage of students expressing the same opinion. The free response areas of the questionnaire were independently analyzed by 2 independent observers, and opinions representing more than 20% of the study population were reported. A p value < 0.05 was considered statistically significant. Statistical analyses were performed using JMP V.10 software (SAS institute, Cary, North Carolina, USA). The datasets used and/or analysed during the current study are available from the corresponding author.

## Results

During the 2016–2017 and 2017–2018 academic years, 415 and 408 second-year undergraduate medical students were enrolled at the Paris-Diderot University, and 414 (99.8%) and 402 (98.5%) students, as well as 102 and 104 educators, participated in the simulation-based education program respectively. In order to cover all the learners, a duration of use of the simulation platform of 28 h/year was required. Thus, 16 educators were simultaneously present on-site, each supervising a small group of no more than 4 students in a dedicated room. The survey was completed by 379 (92%) and 343 (85%) students, as well as 85 (83%) and 104 (100%) educators during the 2016–2017 and 2017–2018 academic years respectively. Therefore, a total of 722 surveys completed by students and 189 surveys completed by educators were analyzed in the present study.

**Fig. 1:**
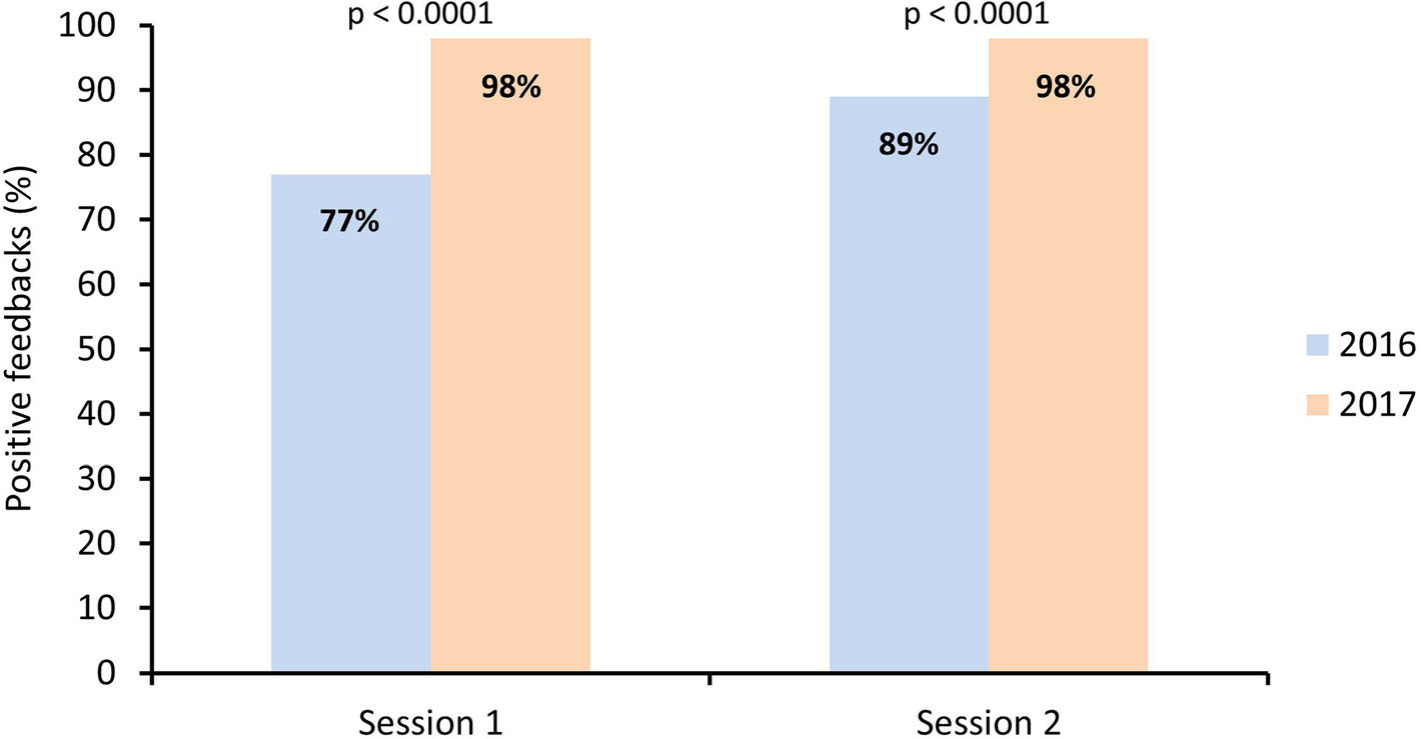
Proportion of overall positive feedbacks by students for both sessions during the 2016–2017 and 2017–2018 academic years

**Fig. 2:**
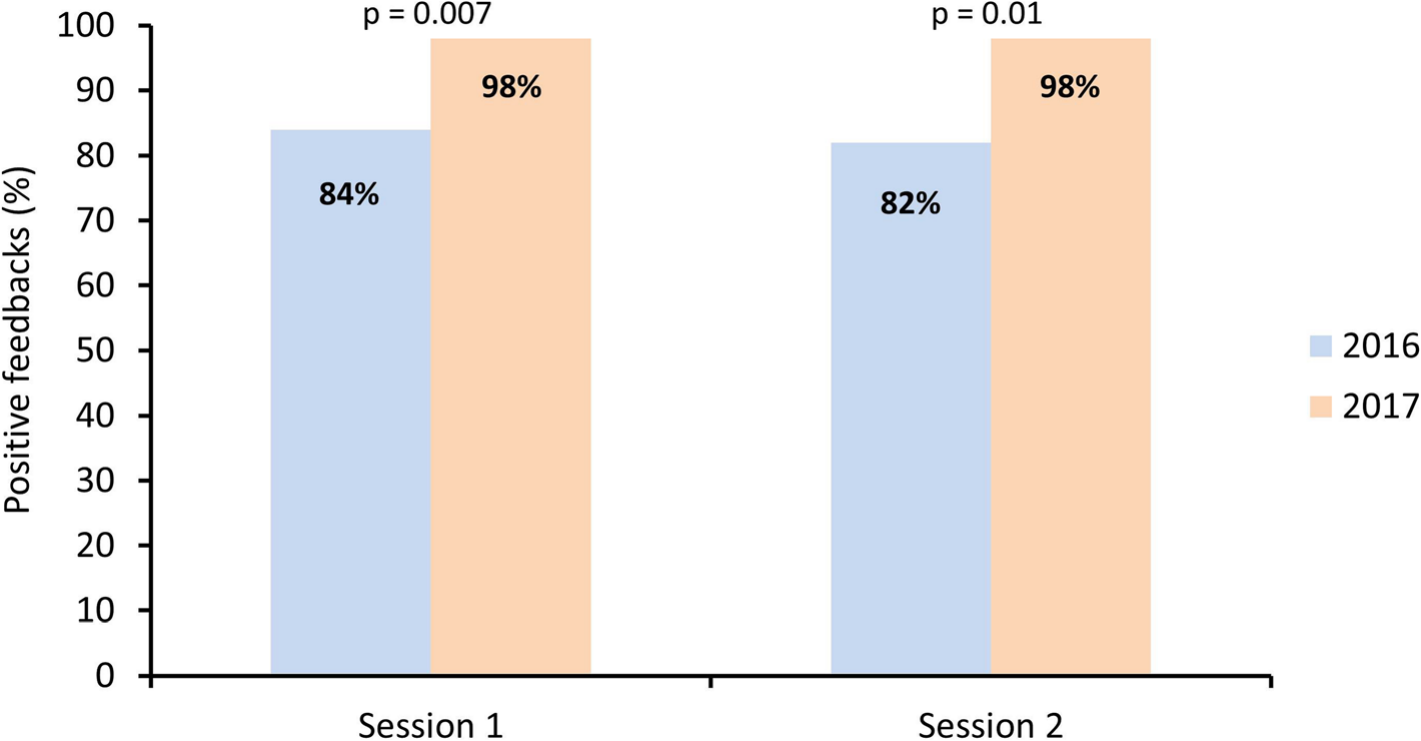
Proportion of overall positive feedbacks by educators for both sessions during the 2016–2017 and 2017–2018 academic years

Students’ overall appreciation improved from the 2016–2017 to the 2017–2018 academic years (Fig. 1). Thus, the appreciation for the first session was considered positive (overall score ≥ 2) by 276 (77%) and 336 (98%) students during the 2016–2017 and 2017–2018 academic years respectively (p < 0.0001). Similarly, the number of positive feedbacks for the second session improved from 325 (89%) to 337 (98%) (p < 0.0001). Educators’ overall satisfaction with the course also improved from 37 (84%) positive appreciations to 55 (98%) for the first session (p = 0.007) and from 34 (82%) to 47 (98%) for the second session (p = 0.01) (Fig. 2).

Feedbacks by students were positive in both years and both sessions regarding the usefulness of the course, fulfillment of pedagogical objectives, quality of the teaching method, time management, educator-student interactivity and relationship (all mean scores ≥2). In contrast, the quality of the simulation equipment was considered as poor the first year and improved the following year (1.43 ± 0.78 vs. 2.11 ± 0.68 respectively, p < 0.0001). Students’ and educators’ feedbacks on the course are presented in Table 1.

**Table 1:** Students’ and educators’ feedbacks on the course

	Session 1			Session 2		
Students’ feedback	2016 (N = 379)	2017 (N = 343)	p	2016 (N = 379)	2017 (N = 343)	p
Usefulness of the course	2.43 ± 0.68	2.76 ± 0.43	< 0.0001	2.43 ± 0.74	2.73 ± 0.48	< 0.0001
Fulfillment of pedagogical objectives	2.24 ± 0.75	2.55 ± 0.53	< 0.0001	2.43 ± 0.62	2.62 ± 0.55	0.002
Quality of the teaching method	2.60 ± 0.57	2.75 ± 0.45	0.001	2.67 ± 0.55	2.76 ± 0.44	0.06
Time management during the session	2.49 ± 0.66	2.55 ± 0.55	0.87	2.58 ± 0.59	2.70 ± 0.48	0.03
Educator-student interactivity and relationship	2.63 ± 0.60	2.89 ± 0.32	< 0.0001	2.69 ± 0.53	2.87 ± 0.37	< 0.0001
Quality of the simulation equipment	1.43 ± 0.78	2.11 ± 0.68	< 0.0001	–	–	–
Total score: overall appreciation	2.31 ± 0.46	2.60 ± 0.33	< 0.0001	2.57 ± 0.46	2.74 ± 0.35	< 0.0001
Educators’ feedback	2016 (N = 44)	2017 (N = 56)	p	2016 (N = 41)	2017 (N = 48)	p
Overall appreciation of the course	1.88 ± 0.42	2.43 ± 0.53	< 0.0001	1.96 ± 0.55	2.23 ± 0.47	0.03

Data are expressed as mean ± SD

The analysis of the free response area of both years and sessions revealed that students most appreciated the auscultation workshop (25%), emphasizing that it allowed studying and comparing normal as well as abnormal heart sounds. Students also acknowledged the practical aspect of the course (22%), with a particular interest in the opportunity of using mannequins for auscultation, measuring blood pressure on each other, and being confronted with short scenarios. Finally, the availability of educators was appreciated (21%), as students highlighted the benefits of working in small groups (25%) that allowed questions to be asked more easily and facilitated educator-student as well as student-student interactivity, in a calmer setting than the hospital. During the first year of implementation, the quality of the simulation material was criticized and considered as the worst aspect of the course by 360 students (95%), mainly because of technical issues. The following year the number of students criticizing the simulation material dropped to 116 (34%).

## Discussion

In this study, we report an original experience regarding the implementation of a large-scale simulation-based cardiovascular diagnostics course for undergraduate medical students. Despite the participation of more than 400 students each year, the implementation proved feasible and successful. We observed a significant improvement from the first to the second year as evidenced by the positive feedbacks of both students and educators.

A major issue encountered during the first year of implementation was related to poor quality simulation mannequins, which represented by far the main complaint by students and educators. Mannequins were considered of poor quality, with heart sounds different from reality and not in line with the national curriculum and the theoretical objectives of the local second-year program. As a consequence, they were completely replaced and refunded the following year and an alternative supplier was used (SAM Basic® with a SimScope® stethoscope, Cardionics, Texas, USA). The new mannequins were tested before the purchase and had the advantage of presenting a wider range of customizable heart sounds of which the characteristics were much closer to reality, leading to the significant improvement of the feedback collected. This point emphasizes the importance of a thorough simulation material selection.

During the first year, the teaching sessions were scheduled mid-year, after the students had started learning the clinical examination by examining real patients in wards. Although the appreciation of students was overall positive, many of them mentioned that the simulation-based teaching program would have been much more useful if it had been scheduled before reaching a real patient’s bedside. Consequently, the sessions were scheduled at the beginning of the following year. A large proportion of students appreciated the possibility to repeat each step of the clinical examination at their own pace without feeling the pressure of facing a real patient, allowing them to gain confidence and acquire knowledge.

Despite the necessity to commit important resources and the poor quality of the mannequins during the first year of implementation, the overall level of satisfaction of the students and educators remained high, suggesting a positive impact of the student- educator relationship during the training. Indeed, most educators were very enthusiastic on the fact that they had a dedicated time for teaching with a restricted number of students, without having to manage multiple non-educational tasks concomitantly as it is often the case during traditional teaching sessions in hospital wards. Reciprocally, students appreciated the opportunity to benefit from the full attention of their educators and emphasized that they could ask questions freely. Thus, the proposed format encouraged the active involvement of all students through each step of the course, as they were constantly questioned on the cardiovascular clinical examination. It has been previously reported that small-group discussion and repeated auscultation of simulated heart sounds improves auscultation proficiency [1, 17].

Although similar teaching programs have been described in literature [7, 18], the implementation of such a large scale program has seldom been reported. We showed that developing such a program is feasible and widely appreciated by students as well as educators. Despite the need to commit significant human and material resources to carry out this educational project, students reacted favorably to the training sessions. Consequently, it can be concluded that Kirkpatrick’s level 1 was reached [15, 16, 19], encouraging us to continue and improve this educational program.

Implementing a simulation-based education program to such a large number of students is a time consuming and difficult task. The development of a dedicated platform is a key factor for success as it allows bringing together educators, students, as well as technical and administrative staffs, in a single facility specially designed to facilitate the use of simulation equipment and to constitute small groups of students. The work accomplished by the administrative staff is of paramount importance to schedule sessions and coordinate the presence of students and educators. In addition, the presence of a competent technical staff on site is crucial to immediately correct major and minor technical issues, so that educators can dedicate all their time to teaching.

Finally, it is worth emphasizing that the overall appreciations were high and improved from one year to the next. This improvement may not only be related to the positive consequences of the replacement of the simulation material, but it may also reflect the learning curve of educators who gained experience over time in using new educational tools.

This study has several limitations [20]. First, the quality of the education program was assessed on the basis of the analysis of educators’ and students’ opinions, and students’ cardiovascular knowledge was not evaluated [21]. However, it is worth emphasizing that the objective of this pilot study was to assess whether level 1 was reached in Kirkpatrick’s model. Second, educators’ opinion evaluation was based on a single question. Third, changing the timing of the sessions, from mid-year to the beginning of the year, may have positively impacted the results of the surveys. Moreover, the implementation of a new educational activity may be associated with increased enthusiasm from both educators and students, and may also have impacted the results of the surveys. Lastly, although the implementation of the simulation-based education program has been a success, the relatively high cost of the simulation material and of creating such a platform, as well as the necessity to recruit a large number of educators, may represent a limit to the generalization of this teaching approach.

## Conclusions

Despite the need to commit significant human and material resources to carry out this large-scale educational project, it was feasible, the students reacted favorably, and Kirkpatrick’s level 1 was reached. Further studies evaluating students’ skills acquisition following the teaching program are necessary to confirm the benefits of this method.

## Data Availability

The datasets generated and/or analyzed during the current study are available from the corresponding author on request.
